# Associations of Physical Activity and Psychological Resilience with Non-Suicidal Self-Injury Among Chinese Adolescents: A Combined Variable- and Person-Centered Approach

**DOI:** 10.3390/bs16050785

**Published:** 2026-05-15

**Authors:** Chang Hu, Wen Zhang, Joston Gary

**Affiliations:** 1 School of Physical Education, Jiangxi Normal University, Nanchang 330022, China; huchang@itc.ynu.edu.cn (C.H.); zhangwen@jxnu.edu.cn (W.Z.); 2 School of Physical Education, Yunnan University, Kunming 650106, China; 3 Department of Management and Engineering, Linköping University, SE-581 83 Linköping, Sweden

**Keywords:** non-suicidal self-injury, physical activity, psychological resilience, latent profile analysis, mediation, adolescents

## Abstract

Non-suicidal self-injury (NSSI) among adolescents is an important public health concern. This study examined the associations among physical activity (PA), psychological resilience (PR), and NSSI among 2257 junior high school students aged 12–17 years in central China. Using both variable-centered and person-centered approaches, the study found that higher PA and higher PR were related to lower NSSI. PR also partly accounted for the association between PA and NSSI, suggesting that resilience may be one pathway linking physical activity to reduced self-injury risk. Latent profile analysis identified three PA–PR profiles: low, moderate, and high. Adolescents in the low PA–PR profile reported the highest NSSI risk, whereas those in the high PA–PR profile reported the lowest risk. These findings suggest that interventions promoting PA and resilience may help reduce adolescent NSSI risk.

## 1. Introduction

Non-suicidal self-injury (NSSI) has become an important public health issue in adolescent mental health worldwide (Lawrence et al., 2023; Ma et al., 2025; W. Yu et al., 2023). Its high prevalence and hidden nature may harm adolescents’ physical and psychological development (Geng et al., 2025; Hu et al., 2026b; W. Zhang et al., 2026; Serafini et al., 2023). NSSI is commonly defined as the intentional and direct damage of one’s own body tissue without suicidal intent, including behaviors such as cutting, burning, and scratching (Y.-J. Wang et al., 2022). Although NSSI does not involve an explicit intention to die, it can cause immediate physical injury and may be associated with later emotional distress and impaired functioning (Faura-Garcia et al., 2024; Hu et al., 2025; Lei et al., 2024; Y. Wang et al., 2022).

Adolescence is a developmental period marked by rapid physical, psychological, and social changes. During this period, difficulties in emotion regulation, peer relationships, academic pressure, and identity development may increase vulnerability to NSSI (Brown & Plener, 2017; X. Li et al., 2024). Epidemiological studies show that approximately 17–33% of adolescents globally have reported at least one NSSI experience (Boduszek et al., 2021; Daukantaitė et al., 2021; Poudel et al., 2022). Beyond being a direct manifestation of personal suffering, NSSI can trigger a chain of negative outcomes, including smoking, alcohol use, social isolation, lack of physical activity (PA), irregular sleep patterns, and even suicidal behavior (Bandel & Brausch, 2020; Fan et al., 2021; Fox et al., 2015; Hepp et al., 2020; Spitzen et al., 2020). Thus, although NSSI is non-fatal or has a low lethality rate, it remains a complex and dangerous public mental health issue. Self-injury behaviors, particularly among adolescents, urgently require increased attention from researchers and stakeholders.

Against this background, this study examines the relationships among PA, psychological resilience (PR), and NSSI among adolescents. PA may be relevant to NSSI because it is closely related to physical health, emotional regulation, and daily behavioral routines. PR is also important because it reflects adolescents’ capacity to adapt to stress and recover from psychological difficulties. Examining PA and PR together may provide a clearer understanding of how health behavior and psychological resources are associated with NSSI.

This study uses both variable-centered and person-centered approaches. The variable-centered approach examines the overall associations among PA, PR, and NSSI, while the person-centered approach identifies subgroups of adolescents with different PA and PR patterns. Combining these two approaches allows the study to capture both general relationships and individual heterogeneity. The findings may provide evidence for developing more targeted prevention strategies for adolescents at risk of NSSI and may help inform school-based and community-based mental health interventions.

## 2. Literature Review and Hypotheses Development

### 2.1. Physical Activity and Non-Suicidal Self-Injury

According to the World Health Organization, physical activity (PA) refers to any bodily movement produced by skeletal muscles that requires energy expenditure, including activities related to work, play, household tasks, transportation, and recreation (WHO, 2010). Beyond its role in physical fitness, PA is closely related to adolescents’ emotional regulation and mental health (Guddal et al., 2019; Hu et al., 2026d; C. Liu et al., 2024). During adolescence, a critical period of physical and psychological development, regular PA can modulate the neuroendocrine system through physiological mechanisms such as enhancing prefrontal cortex functioning and promoting endorphin secretion (Doré et al., 2020; Sorensen & Zarrett, 2014; Wassenaar et al., 2019). These effects not only improve cardiopulmonary function but also serve as protective factors for stress relief and emotional regulation (Gualdi-Russo et al., 2022; Hu et al., 2026a).

Insufficient PA remains common among adolescents worldwide, a large proportion of adolescents do not meet recommended activity levels, with rates reaching 81% (Ashdown-Franks et al., 2019; Ricardo et al., 2022). When adolescents have fewer opportunities for active stress release, their emotion regulation capacity may become more vulnerable under pressure (Loeb et al., 2021). Adolescents with lower levels of PA may show stronger physiological responses to negative emotions, such as higher cortisol reactivity and slower emotional recovery (Bell et al., 2019; Pauly et al., 2019; Wan et al., 2025). Such deficiencies in emotion regulation may then increase the likelihood of maladaptive coping behaviors, including NSSI (Shapero et al., 2019).

PA may also be related to NSSI through social and self-regulatory pathways. Adolescents who participate less in PA may have fewer opportunities for peer interaction, teamwork, and social support (McHale et al., 2022; Prochnow et al., 2020). They may also have fewer experiences of achievement, competence, and perceived control, which are important for self-efficacy and adaptive coping (Sum et al., 2018). When adolescents lack these protective experiences, self-injury may become a maladaptive way to relieve distress, manage emotional pain, or signal psychological needs. Insufficient PA is associated with depression, anxiety, and aggressive behavior, all of which are relevant risk factors for NSSI (Guan et al., 2024; Khazaie et al., 2023; Zhou et al., 2024). Therefore, lower PA may be associated with greater NSSI risk through emotional, social, and behavioral vulnerabilities.

### 2.2. The Mediating Role of Psychological Resilience

PR refers to the capacity to adapt to stress and recover psychological balance under adversity (Schäfer et al., 2024; Troy et al., 2023). Stress-coping theories suggest that mental health outcomes are shaped by the dynamic interaction between individual psychological traits and external behavioral factors (Kang et al., 2023). As a positive psychological resource, PR is associated with lower stress vulnerability and more stable emotional functioning under difficult conditions (Afek et al., 2021; Ungar & Theron, 2020). Within the bio-psycho-social understanding of adolescent NSSI, resilience has been identified as a protective factor because it is related to more adaptive coping, better emotion regulation, and lower self-injury risk (Stefanovics et al., 2023).

PA may contribute to PR by providing adolescents with repeated experiences of physical effort, goal pursuit, self-control, and social interaction. Adolescents with higher PA tend to report stronger self-efficacy, better coping ability, and more adaptive emotion regulation (Gerber et al., 2012). PA has also been associated with neurobiological and psychosocial processes that are relevant to resilience, including improved neuroendocrine functioning and stronger social connectedness (Chen & Nakagawa, 2023; Jarvi et al., 2017). These findings suggest that PA may help adolescents build psychological resources that support adaptation under stress. 

PR is also closely related to NSSI. Adolescents with higher PR are more likely to tolerate distress, regulate negative affect, and use personal and social resources when facing difficulties. In contrast, lower PR may increase emotional dysregulation and make maladaptive coping behaviors more likely. Since NSSI is often used to regulate intense emotional pain, weaker resilience may increase adolescents’ vulnerability to self-injury.

Although previous studies have examined the relationship between PA and PR, and between PR and NSSI, these relationships have rarely been tested within the same explanatory framework. PR may serve as a pathway through which PA is associated with lower NSSI. In this study, PR is therefore examined as a mediating mechanism linking PA to NSSI.

### 2.3. The Person-Centered Perspective

Some studies on PA, PR, and NSSI have used variable-centered methods, which estimate average associations across the full sample. This approach is useful for identifying general relationships among variables, but it may hide meaningful differences among individuals (Spurk et al., 2020). Adolescents may differ not only in their levels of PA or PR, but also in how these two characteristics combine within the same person. For this reason, a single average effect may not describe the association between PA, PR, and NSSI across adolescent subgroups.

Person-centered methods, such as latent class analysis and latent profile analysis (LPA), are designed to identify subgroups of individuals with similar patterns across multiple variables (He & Fan, 2019). LPA can classify adolescents according to their combined levels of PA and PR, allowing researchers to examine whether different PA–PR profiles are associated with different levels of NSSI (Huang et al., 2025; Yuan et al., 2025). This approach does not replace variable-centered analysis. Instead, it adds information about heterogeneity that average associations cannot show. At present, limited research has examined how combined patterns of PA and PR relate to NSSI among adolescents. Therefore, a person-centered approach provide a more detailed understanding of which adolescents show higher or lower NSSI risk.

### 2.4. Current Study and Hypotheses

The study integrates variable-centered and person-centered approaches to examine the relationships among PA, PR, and NSSI in adolescents. From a variable-centered perspective, the study tests whether PA is negatively associated with NSSI and whether PR mediates this association. From a person-centered perspective, the study uses LPA to identify subgroups of adolescents with different PA and PR patterns and then compares NSSI across these profiles. Combining these two approaches allows the study to examine both the general association among PA, PR, and NSSI and the heterogeneity across adolescent subgroups.

By positioning PR as a mediating mechanism, this study examines one possible pathway through which PA may be linked to lower NSSI. By using LPA, the study further considers whether adolescents with different combinations of PA and PR differ in their NSSI risk. This design may provide evidence for prevention strategies that consider both health behavior and psychological resilience.

**Hypothesis** **1.** *PA is negatively associated with adolescent NSSI.*

**Hypothesis** **2.** *PR mediates the association between PA and NSSI.*

**Hypothesis** **3.** *Distinct latent combinations of PA and PR exist among adolescents, and these different profiles are associated with significant differences in NSSI.*

## 3. Materials and Methods

### 3.1. Participants and Procedure

This study was approved by the Ethics Committee of Jiangxi Normal University (Protocol Code: IRB-JXNU-PEC-20240103). All procedures followed the Declaration of Helsinki and relevant regulations in China. Participation was voluntary. Before data collection, written informed consent was obtained from both students and their legal guardians. Participants were informed of the study purpose, the anonymous and confidential nature of the survey, and their right to withdraw at any time without penalty.

Before the survey, all investigators received standardized training on questionnaire administration. The survey was conducted in classroom settings with support from schools‘ administrators and teachers. Questionnaires were completed within approximately 20 to 30 min. Students were instructed to complete the questionnaires independently, and completed questionnaires were collected and checked on site.

A mixed sampling strategy combining stratified sampling and cluster sampling was used. Cities in a central province of China were first stratified into three economic levels, high, medium, and low, based on regional gross domestic product (GDP) per capita and official statistical reports. One city from each stratum was then selected according to accessibility, feasibility of data collection, and cooperation with local education authorities.

Within each selected city, schools were recruited using cluster sampling. One urban and one rural middle school were selected in each city according to administrative classifications provided by the local education bureau, resulting in 12 schools. Within each school, one to three intact classes from Grades 7 to 9 were selected based on school scheduling and administrative coordination. All students present in the selected classes on the day of data collection were invited to participate.

A total of 2680 questionnaires were distributed and returned. During data screening, responses were excluded if they showed patterned or uniform answering, failed embedded validity check items, or contained more than 15% missing data. These criteria were applied at the questionnaire level, and the full questionnaire was excluded if any criterion was met. After screening, 2257 valid questionnaires were retained, yielding an effective response rate of 84.22%.

For the retained sample, missing data were low, with less than 5% missingness for each variable. Missing values were handled using item-level mean imputation. Given the low level of missingness, more complex methods such as multiple imputation were not used. This approach is consistent with prior research suggesting that simple imputation may be acceptable when the proportion of missing data is small (Hu et al., 2026c).

Participants ranged in age from 12 to 17 years (M = 13.55, SD = 1.21). The final sample included 998 urban students (44.2%), 1259 rural students (55.8%), and 991 males (43.9%) and 1266 females (56.1%). In terms of grade distribution, 865 students (38.3%) were in Grade 7, 844 (37.4%) were in Grade 8, and 548 (24.3%) were in Grade 9.

Early to middle adolescence is an important developmental period for studying NSSI because emotion regulation capacities are still developing and vulnerability to self-injury may begin to emerge. Focusing on middle school students therefore helps identify early risk patterns and related psychosocial factors.

### 3.2. Measures

#### 3.2.1. Physical Activity

PA was assessed using the revised Physical Activity Rating Scale (PARS-3), originally developed by Hashimoto and widely used in Chinese populations (G. Yang et al., 2021; W. Zhang et al., 2026). The scale consists of three items that assess an individual’s exercise intensity, duration, and frequency. Each dimension is rated on a 5-point Likert scale (1–5). For example, exercise intensity ranges from “light activity (e.g., walking)” to “high-intensity activity (e.g., fast running or competitive sports)”; duration ranges from “less than 10 min” to “more than 60 min”; and frequency ranges from “rarely or no participation” to “multiple times per week or almost daily.” It should be noted that the scale does not have a multi-item subscale structure; a single item measures each dimension. The total physical activity score is calculated using the formula: intensity × (duration − 1) × frequency, with a range of 0 to 100. Higher scores indicate higher levels of physical activity. The scale was used to assess participants’ physical activity over the past month. Existing research has shown that the PARS-3 has good reliability and validity. In this study, the internal consistency coefficient of the scale was Cronbach’s α = 0.74.

#### 3.2.2. Non-Suicidal Self-Injury

NSSI was assessed using the Ottawa Self-Injury Inventory (OSI) developed by Nixon et al. (2015). The scale includes 10 items measuring different forms of self-injurious behavior, such as head banging, severe self-biting, skin scratching or picking, and cutting or stabbing with sharp objects. Participants were asked to report the frequency of each behavior during the past year using a 4-point scale: 0 = never, 1 = once, 2 = 2–4 times, and 3 = 5 or more times. A total NSSI score was calculated by summing the item scores, with higher scores indicating greater severity of NSSI. In addition, consistent with prior studies using the OSI, participants who reported any self-injurious behavior (i.e., any item scored > 0) were classified as engaging in NSSI. This binary indicator was used to describe the prevalence of NSSI in the sample, whereas the continuous total score was retained for subsequent statistical analyses to capture NSSI severity variability. The OSI has good psychometric properties, including satisfactory internal consistency, construct validity, and convergent validity in adolescent samples (Guo et al., 2024; Lutz et al., 2024). In the present study, the OSI demonstrated good internal consistency, with a Cronbach’s α of 0.85.

#### 3.2.3. Psychological Resilience

PR was measured using the 10-item Connor-Davidson Resilience Scale (CD-RISC-10) developed by Campbell-Sills and Stein (2007). This abbreviated scale assesses an individual’s resilience over the past month. Each item is rated on a 5-point Likert scale ranging from 0 (never) to 4 (always), with higher scores indicating greater frequency of resilient responses. For example, items include statements such as “I am able to adapt when changes occur” and “I tend to bounce back after illness or hardship.” The total score is obtained by summing all item responses, with higher scores indicating greater psychological resilience. The CD-RISC-10 has good psychometric properties across diverse populations, including adolescents, with strong internal consistency and well-established construct validity (Lyu & Chen, 2026; Mohammad et al., 2023). In the present study, the CD-RISC-10 demonstrated good internal consistency (Cronbach’s α = 0.87).

### 3.3. Data Analysis

Data were analyzed using SPSS 26.0 and Mplus 8.3. Prior to the main analyses, missing data were examined and handled as described above. Assumptions for parametric analyses were also assessed. Normality was evaluated using skewness and kurtosis indices, and all variables fell within acceptable ranges. Multicollinearity was examined using variance inflation factors (VIFs), with all values below the recommended threshold of 5, indicating no serious multicollinearity. First, descriptive statistics and Pearson correlation analyses were conducted in SPSS 26.0 to examine the distributions and associations among the main study variables. Internal consistency reliability for each scale was assessed using Cronbach’s α. Second, to test the mediating effect proposed in Hypothesis 2, PROCESS 3.5 (Model 4) was employed. Indirect effects were estimated using a bootstrapping procedure with 5000 resamples, and 95% confidence intervals (CIs) were calculated.

Third, to address the study’s person-centered objective (Hypothesis 3), latent profile analysis (LPA) was conducted in Mplus 8.3 using adolescents’ PA and PR scores. Model selection was evaluated using multiple fit indices, including the Akaike Information Criterion (AIC), Bayesian Information Criterion (BIC), and sample-size-adjusted BIC (aBIC), with lower values indicating better model fit. In addition, the Lo-Mendell-Rubin likelihood ratio test (LMR) and the bootstrap likelihood ratio test (BLRT) were used to compare models, with a *p*-value less than 0.05 indicating that the current model fit the data significantly better than the model with one fewer class (He & Fan, 2019). Entropy values greater than 0.80 were considered indicative of good classification accuracy. These criteria were considered together to determine the optimal number of latent profiles.

Finally, one-way analysis of variance (ANOVA) was conducted to examine differences in NSSI scores across the identified latent profiles. The significance level for all analyses was set at *p* < 0.05.

## 4. Results

### 4.1. Variable-Centered Approach

#### 4.1.1. Associations Among PA, PR, and NSSI

Descriptive statistics and correlation results are presented in Table 1. As expected, PA was positively associated with PR (r = 0.551, *p* < 0.01). In addition, PA was negatively associated with NSSI (r = −0.457, *p* < 0.01), and PR was also negatively associated with NSSI (r = −0.522, *p* < 0.01). These patterns of associations are consistent with Hypothesis 1.

Furthermore, the absolute values of skewness and kurtosis for all variables were below 3, suggesting that the assumption of approximate normality was met (Mardia, 1970).

#### 4.1.2. Mediation Analysis

From a variable-centered perspective, this study examined the relationships among PA, PR, and NSSI and tested the mediating role of PR. After standardizing all variables, NSSI was entered as the dependent variable, PA as the independent variable, PR as the mediator, and gender and household registration as control variables. Mediation analysis was conducted using PROCESS 3.5 (Model 4).

**Figure 1:**
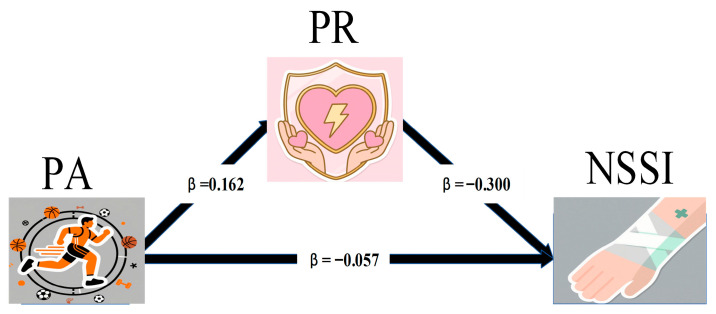
Mediation Pathway.

The results showed that PA was negatively associated with NSSI (*β* = −0.105, *p* < 0.001) and positively associated with PR (*β* = 0.162, *p* < 0.001). When both PA and PR were included in the model, PA remained negatively associated with NSSI (*β* = −0.057, *p* < 0.001), while PR was also negatively associated with NSSI (*β* = −0.300, *p* < 0.001) (see Table 2 and Figure 1).

Bootstrap analysis with 5000 resamples indicated that the indirect effect was statistically significant (indirect effect = −0.049, 95% CI [−0.055, −0.042]) (see Table 3). The indirect effect accounted for approximately 46% of the total effect, suggesting that a substantial proportion of the association between PA and NSSI was mediated by PR.

### 4.2. Person-Centered Results

#### 4.2.1. Latent Profiles of PA and PR

From a person-centered perspective, LPA was conducted to identify subgroups based on PA and PR. Models specifying one to five profiles were estimated sequentially in Mplus 8.3 using the 13 observed indicators. Model fit was evaluated using multiple criteria, including AIC, BIC, and aBIC, with lower values indicating better fit. As the number of profiles increased, these indices decreased progressively; however, the rate of improvement diminished after the three-profile solution, suggesting limited incremental gain in model fit. In addition, the LMR test was significant across all models, indicating that models with more profiles fit better than those with fewer profiles.

Although the five-profile solution yielded the highest entropy, several classes in this model accounted for fewer than 5% of the total sample, raising concerns about stability and interpretability. In contrast, the three-profile solution demonstrated a favorable balance between statistical fit, classification accuracy, and conceptual interpretability.

Therefore, based on these criteria, the three-profile solution was selected as the optimal model. Detailed model fit indices are presented in Table 4.

**Figure 2:**
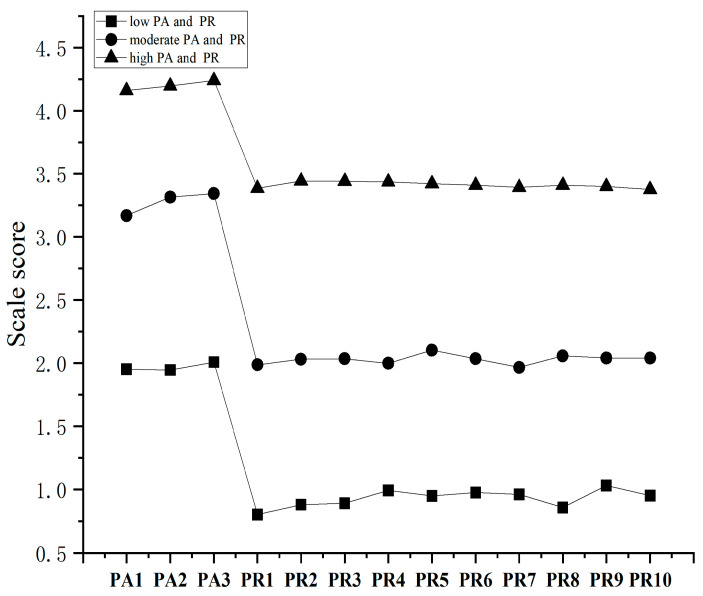
Score Distributions.

Figure 2 illustrates the distinct latent profiles of PA and PR. The three-profile solution revealed meaningful heterogeneity in the combined patterns of PA and PR, with each profile characterized by a distinct configuration of mean scores across indicators. Based on the observed score distributions and consistent with prior research (Ma et al., 2025), the profiles were labeled as follows. The first profile (C1: Low PA and PR; 16.70%) was characterized by consistently low scores across all indicators, reflecting relatively limited engagement in PA and lower levels of PR. The second profile (C2: Moderate PA and PR; 54.23%) exhibited moderate scores across indicators, suggesting a relatively balanced pattern of PA and PR. The third profile (C3: High PA and PR; 29.07%) showed consistently high scores across indicators, indicating higher levels of PA engagement alongside elevated PR. These profiles capture gradations in the co-occurrence of PA and PR rather than isolated differences in either construct.

#### 4.2.2. Differences in NSSI Scores Across Latent Classes

As indicated by Table 5, there are significant differences in NSSI scores among adolescents across different latent classes (*F* = 185.554, *p* < 0.001). Post hoc tests revealed that the NSSI scores follow the order: C1 > C2 > C3. These findings highlight the heterogeneity in PA and PR among adolescents, with distinct latent classes showing marked differentiation in NSSI.

## 5. Discussion

This study examined the associations among physical activity (PA), psychological resilience (PR), and non-suicidal self-injury (NSSI) in adolescents using both variable-centered and person-centered approaches. The variable-centered analysis tested the association between PA and NSSI and examined whether PR served as a mediating mechanism. The person-centered analysis used latent profile analysis to identify subgroups of adolescents with different PA and PR patterns.

### 5.1. Variable-Centered Approach

The variable-centered analysis showed that PA was negatively associated with NSSI among adolescents, which is consistent with previous studies and supports Hypothesis 1 (Lai et al., 2024; Zhou et al., 2024). This finding can be understood through emotion regulation theory. Regular PA may help adolescents regulate negative emotions by supporting physiological processes related to mood and stress, including the secretion of endorphins and other neurochemical processes involved in emotional regulation (Biddle et al., 2019; L. Liu et al., 2024; T. Wang et al., 2024). When adolescents have insufficient PA, they may have fewer opportunities to release stress through adaptive bodily activity. Under academic pressure, peer conflict, or family stress, this may make negative emotions more difficult to manage (Gross, 2015; McRae & Gross, 2020). In this context, NSSI may become a maladaptive way to reduce emotional distress or regain a sense of control (Gao et al., 2024; Z. Li et al., 2024; G. Zhang et al., 2024).

Social support theory helps explain the association between PA and adolescent NSSI (Shumaker & Brownell, 1984). PA often occurs in social contexts, such as school sports, group exercise, or informal peer activities. These contexts provide opportunities for adolescents to build relationships, receive peer support, and share emotional experiences (Mitic et al., 2021). By contrast, low PA may be linked to a more sedentary lifestyle and fewer opportunities for social interaction (Omorou et al., 2020). A When adolescents experience loneliness, depressive symptoms, or limited social support, they may be more likely to use self-injury as a maladaptive coping behavior (Hoare et al., 2016; Kandola et al., 2020; Rodriguez-Ayllon et al., 2019; Vancampfort et al., 2018). Thus, PA may be associated with lower NSSI not only through physiological regulation, but also through social connection.

The mediation analysis showed that PR mediated the association between PA and NSSI, supporting Hypothesis 2. This result suggests that PA may be linked to lower NSSI partly by strengthening adolescents’ psychological resources. Regular PA can provide experiences of effort, persistence, mastery, and goal attainment. These experiences may increase self-efficacy, frustration tolerance, and perceived competence, which are important components of resilience (Ran et al., 2022; Tian et al., 2021; Wei et al., 2022). Higher PR may then help adolescents cope more adaptively with stress, regulate negative affect, and use personal and social resources when facing difficulties (Arslan, 2016; Xiao et al., 2020; Meng et al., 2022; Q. Yang et al., 2023). In contrast, lower PR may intensify emotional distress under stress and increase the likelihood of maladaptive coping behaviors, including NSSI (Khazaie et al., 2023; Zhou et al., 2024; Zhu et al., 2020). These findings indicate that PR is an important psychological pathway linking PA to adolescent NSSI.

### 5.2. Person-Centered Approach

Building on the variable-centered findings, this study further adopted a person-centered approach to capture heterogeneity within the adolescent sample. The latent profile analysis identified three subgroups: Low PA and PR (16.70%), Moderate PA and PR (54.23%), and High PA and PR (29.07%). The Low PA and PR subgroup showed low levels of both PA and PR, the Moderate PA and PR subgroup showed intermediate levels of both variables, and the High PA and PR subgroup showed high levels of both variables. These profiles indicate that adolescents differ not only in each protective factor separately, but also in how PA and PR are combined within individuals.

NSSI differed across the three profiles. Adolescents in the Low PA and PR subgroup reported the highest NSSI scores, those in the Moderate PA and PR subgroup reported lower scores, and those in the High PA and PR subgroup reported the lowest scores. The mediation results and suggests that PA and PR may function together as protective factors. Insufficient PA may limit opportunities for physiological stress regulation, emotional release, and social interaction. Low PR may further reduce adolescents’ ability to cope with adversity and regulate negative affect. When these two vulnerabilities occur together, adolescents may have fewer adaptive resources for managing distress and may become more vulnerable to NSSI (James et al., 2024; H. Yu et al., 2023).

By contrast, adolescents in the High PA and PR subgroup may benefit from both behavioral and psychological resources. Regular PA may support physical functioning, emotional regulation, social connection, and self-efficacy. Higher PR may strengthen adaptive coping, frustration tolerance, and the ability to seek or use support under stress. The co-occurrence of high PA and high PR may therefore create a more favorable resource pattern for adolescents facing emotional difficulties.

The person-centered findings also have practical implications for prevention. The three profiles may help schools and community services identify adolescents with different levels of need. Adolescents in the High PA and PR subgroup may benefit from universal prevention programs that maintain regular PA participation and support socio-emotional skills, including emotion regulation, peer interaction, and stress awareness. Adolescents in the Moderate PA and PR subgroup may benefit from selective group-based programs that combine PA with coping skills, problem-solving, and peer support. Adolescents in the Low PA and PR subgroup may require more intensive support, such as individual or small-group programs that combine PA promotion with stress management, emotion regulation training, counseling, and efforts to reduce barriers to PA participation. These differentiation suggest that PA promotion may be more useful when combined with resilience-building components and adjusted to adolescents’ profile characteristics.

### 5.3. Limitations

Several limitations should be considered. First, the sample included only junior high school students from 12 middle schools in one central province of China. Although the study used a stratified sampling strategy, it did not include adolescents from other regions, senior high schools, or more diverse sociocultural backgrounds. Therefore, the findings should be generalized with caution. In addition, the study was based on a community sample rather than a clinically identified high-risk sample. The results may not fully apply to adolescents with severe or persistent self-injurious behaviors. Future studies should use larger, multi-regional, and more diverse samples to improve external validity.

Second, the cross-sectional design limits causal interpretation. Although the results were consistent with the proposed associations among physical activity (PA), psychological resilience (PR), and non-suicidal self-injury (NSSI), the temporal order of these variables cannot be determined. Lower PA and PR may be related to higher NSSI risk, but adolescents with more frequent or severe NSSI may also be less likely to engage in PA or maintain higher resilience. Longitudinal and novel studies are needed to examine causal pathways and developmental changes over time.

Third, all variables were measured through self-report questionnaires, which may introduce recall bias, social desirability bias, and common method variance. This concern is especially relevant for NSSI, because adolescents may underreport sensitive behaviors. Future research could improve measurement validity by using multiple informants, behavioral observations, objective PA measures, such as accelerometers or wearable devices, and clinical interviews. In addition, PR was measured as a global construct using the CD-RISC-10. Future studies could examine specific dimensions of resilience to determine which aspects of PR are most closely related to NSSI.

Fourth, although the latent profile analysis identified three PA–PR profiles, the stability and generalizability of these profiles require further testing. Latent profile solutions may vary across samples, so future studies should examine whether the same profile structure can be replicated in independent samples. The present study also did not examine changes in profile membership over time. Longitudinal person-centered methods, such as latent transition analysis, could help clarify how changes in PA and PR profiles are related to changes in NSSI risk.

Fifth, the analyses may not have fully captured contextual and structural variation. Students were nested within classes and schools, but this hierarchical structure was not explicitly modeled. Unobserved class- or school-level factors may therefore have influenced the estimates. In addition, although several demographic variables were controlled in the mediation analysis, other relevant factors were not included, such as family functioning, socioeconomic background, depressive symptoms, anxiety, peer relationships, and stressful life events. Future studies should consider multilevel designs and include a broader set of psychosocial covariates.

Finally, this study examined PR as the only mediating mechanism. NSSI is shaped by multiple psychological, family, peer, and school-related factors, so the associations observed in this study are likely part of a broader psychosocial process. Future research could extend the model by examining additional mediators and moderators, such as perceived social support, self-efficacy, emotion regulation, family environment, and depressive symptoms. Such work would provide a clearer explanation of how PA may be associated with NSSI among adolescents.

## 6. Conclusions

This study showed that PA was positively associated with PR and negatively associated with adolescent NSSI. Moreover, PR significantly mediated the relationship between PA and NSSI. From a person-centered perspective, three latent profiles of PA and PR were identified, and adolescents in different profiles showed significant differences in NSSI risk. These findings highlight the joint protective roles of PA and PR in adolescent NSSI.

## Figures and Tables

**Table 1:** Descriptive and Correlation Analysis (*n* = 2257).

Variables	1	2	3
1. PA	1		
2. PR	0.551 **	1	
3. NSSI	−0.457 **	−0.522 **	1
*M*	34.355	22.508	11.192
*SD*	31.610	9.327	7.329
Skewness	0.514	−0.089	−0.194
Kurtosis	−1.109	−0.790	−1.014

Note: ** Significantly correlated at the 0.01 level (two-tailed).

**Table 2:** Regression Analysis of PA, PR, and NSSI.

	Model 1 PR		Model 2 NSSI		Model 3 NSSI	
	*β*	*t*	*β*	*t*	*β*	*t*
Gender	−0.865	−2.613 **	1.319	4.782 ***	1.060	4.111 ***
Household Registration	−0.460	−1.392	0.572	2.076 *	0.434	1.687
PA	0.162	31.219 ***	−0.105	−24.329 ***	−0.057	−11.725 ***
PR					−0.300	−18.299
*R*	0.553		0.468		0.566	
*R* ^2^	0.306		0.219		0.320	
*F*	331.220 ***		210.922 ***		265.340 ***	

Notes: * Significant correlation at 0.05 level, ** Significant correlation at 0.01 level, *** Significant correlation at 0.001 level.

**Table 3:** Mediation Effect Analysis.

Pathway	Effect	Boot SE	95%CL		Proportion of Effect
			LLCI	ULCI	
Total	−0.105	0.004	−0.114	−0.097	100%
PA→NSSI	−0.057	0.005	−0.066	−0.047	54%
PA→PR→NSSI	−0.049	0.003	−0.055	−0.042	46%

**Table 4:** Model Fit Indices for Latent Profile Analysis.

Model	AIC	BIC	aBIC	LMR(*p*)	BLRT(*p*)	Entropy	Attribution Probability
Class 1	102,873.942	103,022.708	102,940.102	/	/	/	100
Class 2	96,040.751	96,269.623	96,142.536	<0.001	<0.001	0.869	60.48/39.52
Class 3	94,065.218	94,374.194	94,202.627	<0.001	<0.001	0.883	16.70/54.23/29.07
Class 4	93,332.269	93,721.35	93,505.303	<0.01	<0.001	0.879	23.93/36.07/12.89/27.11
Class 5	92,873.249	93,342.436	93,081.908	<0.01	<0.001	0.895	8.56/24.28/36.28/4.17/26.71

**Table 5:** Differences in NSSI Scores.

Variable	Latent Class (M ± SD)			*F*	Back Testing
	Class 1 (312)	Class 2 (1281)	Class 3 (664)		
NSSI	17.91 ± 5.41	10.57 ± 6.52	9.23 ± 7.81	185.554 ***	1 > 2 > 3

Note: *** Significant correlation at 0.001 level.

## Data Availability

The data used in this study are available from the corresponding author upon reasonable request.

## Abbreviations

The following abbreviations are used in this manuscript:

NSSI	Non-suicidal Self-injury
PA	Physical Activity
PR	Psychological Resilience
LPA	Latent Profile Analysis
AIC	Akaike Information Criterion
BIC	Bayesian Information Criterion
aBIC	Sample Size-Adjusted Bayesian Information Criterion
LMR-LRT	Lo-Mendell-Rubin Likelihood Ratio Test
BLRT	Bootstrap Likelihood Ratio Test
